# Identification of RNA-binding protein YBX3 as an oncogene in clear cell renal cell carcinoma

**DOI:** 10.1007/s10142-023-01145-6

**Published:** 2023-07-07

**Authors:** Chen Wang, Zhijie You, Yihui He, Xin Chen

**Affiliations:** 1 Department of Pathology, Shengli Clinical Medical College of Fujian Medical University, Fujian Provincial Hospital, Fuzhou, 350001 Fujian China; 2 Department of Pathology, Fujian Provincial Hospital South Branch, Fuzhou, 350001 Fujian China

**Keywords:** YBX3, Survival, Immune infiltration, Proliferation, Apoptosis, Carcinoma

## Abstract

Y box binding protein 3 (YBX3) is an indispensable factor for protein synthesis, cellular growth, and proliferation, and is intricately involved in the progression of diverse tumor types. The objective of the current study was to investigate the role of YBX3 in the prognosis, immune infiltration, and progression of clear cell renal clear cell carcinoma (ccRCC). The expression level of YBX3 in ccRCC tissues was compared using The Cancer Genome Atlas (TCGA) and analyzed using the Wilcoxon rank sum test. Logistic regression and multivariate Cox analyses were subsequently employed to scrutinize the association between YBX3 expression and the clinicopathological characteristics of patients. The TIMER 2.0 tool was also utilized to quantify the degree of immune cell infiltration of YBX3. Kaplan Meier analysis was performed to assess the correlation between YBX3 and the survival rate. A high expression level of YBX3 was significantly correlated with the tumor pathological stage, histological grade, TNM stage, and the abundance of aDC, pDC, Th1, and Treg immune cells. Higher expression of YBX3 in advanced ccRCC was found to be associated with a lower overall survival rate in the M0, N0, and T2 subgroups. In vitro, after the silencing of YBX3 in A498 cells and overexpression of YBX3 in ACHN cells, cell proliferation, colony formation, migration, invasion, cell cycle assays, and flow cytometric apoptotic analysis were performed to evaluate the role of YBX3 in the progression of ccRCC. YBX3 was found to be intricately associated with the progression and prognosis of ccRCC, and may serve as an effective treatment target for ccRCC or a biomarker for prognosis prediction.

**Supplementary Information:**

The online version contains supplementary material available at 10.1007/s10142-023-01145-6.

## Introduction

Clear cell renal cell carcinoma (ccRCC) accounts for ~ 85% of all renal cancers (Miller et al. 2019). At present, surgery is still the main treatment for ccRCC (Jonasch et al. 2021). However, in most cases, ccRCC lacks effective early diagnostic indicators and biomarkers for prognostic prediction (Cui et al. 2020; Lv et al. 2023). Therefore, the early identification of effective therapeutic targets or biomarkers for ccRCC would prove to be helpful for the early diagnosis of ccRCC, and the search for potential effective therapeutic targets or biomarkers for ccRCC is urgently required.

The YBX protein family exhibited remarkable sequence homology across diverse species. This family comprised three members, namely YBX1, YBX2, and Y box protein 3 (YBX3). Of these, YBX3 belonged to the primary group of human cold shock domain proteins that regulated various binding proteins (Cooke et al. 2019; Luciani et al. 2018). YBX3, also known as DNA binding protein A (DBPA), zonula occludens 1-associated nucleic acid binding protein (ZONAB), or cold shock domain protein A (CSDA), was an epithelial-specific transcription factor that is ubiquitously expressed in various cell types. It was associated with DNA repair, cell proliferation, and the progression of diverse human ailments (Qin et al. 2020). Recently, mounting evidence has demonstrated that YBX3 was upregulated in various tumor cells. For instance, YBX3 promoted the metastasis of nasopharyngeal carcinoma via PI3K/AKT signaling (Fan et al. 2021). Additionally, YBX3 activation of Kirsten rat sarcoma viral oncogenes attenuated breast cancer progression (Qin et al. 2021). Moreover, researchers (Huang et al. 2020) found that the level of YBX3 was significantly correlated with the overall survival rate and metastasis of hepatocellular carcinoma. It could be employed as a candidate protein standard for inflammation-induced hepatocarcinogenesis. In another study, Xiang and other colleagues (Xiang et al. 2020) utilized LASSO regression and Cox survival analysis to construct a risk model. They found that YBX3 may serve as an independent and accurate prognostic gene in ccRCC. However, further elucidation is required to determine the specific role of YBX3 in ccRCC progression.

## Material and Methods

### Data source and preprocessing

The level 3 genomic data, as well as associated clinical information from the ccRCC project [including 72 normal and 539 tumor tissues; workflow type: HTSeq-Fragments Per Kilobase per Million (FPKM)] were obtained from TCGA (https://portal.gdc.cancer.gov/) on July 27, 2021. The exclusion criteria were normal ccRCC samples and an overall survival less than 30 days. Subsequently, the level 3 ccRCC-HTSeq-FPKM data were converted into transcripts per million reads (TPM) using R package (version 3.6.3), and log2 conversion was subsequently performed for further analyses.

### YBX3 differential expression in pan-cancer and ccRCC tissues in the TCGA database

ccRCC or normal tissues were the source of the variable data used to generate box plots to assess the differential expression of YBX3 in pan-cancer tissues and ccRCC tissues. Paired plots were used to count the differential expression of YBX3 in ccRCC samples and matched paracancerous samples.

### qRT-PCR analysis of the expression level of YBX3 in ACHN, A498 and HK-2 cells.

After total RNA had been extracted from the cells, the expression of YBX3 was quantified using M-MLV-RTase (Promega Corporation, #M1701), and GAPDH was used as an internal control. RNA extraction was performed according to the protocol of TRIzol® Plus RNA Purification Kit (Invitrogen, #12,183–555), cDNA preparation was performed according to the protocol of PrimeScript™ RT Master Mix (Perfect Real Time) (TAKARA, #RR036A) and real-time PCR was performed according to the protocol of TB Green Premix Ex Taq II (Tli RNaseH Plus) (TAKARA, #RR820A). The expression of YBX3 mRNA was detected using StepOnePlus Real-Time PCR System (ABI, USA) with the following program: 95˚C for 30 s, 40 cycles of 95 °C for 5 s, and 60 °C for 30 s. The primers used in the present study, and their sequences, were as follows: for YBX3: 5’-ACCGGCGTCCCTACAATTAC-3’ (forward) and 5’-GGTTCTCAGTTGGTGCTTCAC-3’ (reverse); and for GAPDH: 5’-AGAAGGCTGGGGCTCATTTG − 3’ (forward) and 5’-AGGGGCCATCCACA GTCTTC-3’ (reverse). GAPDH was used as an internal reference gene. The data were analyzed using the 2^−ΔΔCT^ method. 3 technical replicates were performed for each PCR reaction.

### Analysis of the correlation between YBX3 expression and immune infiltration

The infiltration of immune cells (such as aDC, pDC, Th1 and Treg cells) fulfills a vital role in tumor immunotherapy, and improving immune effector cell infiltration may cause significant improvements in the effectiveness of immunotherapy (Zhang et al. 2019). The relative tumor infiltration levels of 24 types of immune cell were quantified using single sample gene set enrichment analysis (ssGSEA) analysis to interrogate the expression levels of genes in published signature gene lists. This immune infiltration algorithm was a built-in algorithm for GSVA packages (Hanzelmann et al. 2013). To explore the correlation between YBX3 and the infiltration levels of immune cells, and the association of infiltration of immune cells with the different expression groups of YBX3, the Wilcoxon rank sum test and Spearman correlation analysis were adopted. In terms of the results, lollipop data illustrations, group comparison charts and scatterplots were utilized to show visualized data.

### Analysis of the association between YBX3 expression and clinicopathological characteristics

All clinical statistical analyses were performed using R package (version 3.6.3), and ggplot2 (version 3.3.3) was employed for visualization of the data. The high or low expressions in each stage were grouped according to the median expression of YBX3, and thus the theoretical frequency table of each stage was established. In analyzing the association between clinicopathological variables and YBX3, the Wilcoxon signed rank test and logistic regression were adopted. All statistical tests were two-sided, and P values < 0.05 were considered to indicate significant values.

### Analysis of the association between YBX3 expression and prognosis

Analysis of OS rates was conducted using Kaplan–Meier plots. First, RNA-seq data in the level 3 HTSeq-FPKM format from the TCGA-KIRC project were converted into the TPM format, and log2 conversion was further performed. Secondly, the R survival package (version 3.2–10; https://cran.r-project.org/web/packages/survival/) was employed for statistical analysis of survival data, and R survminer package (version 0.4.9; https://cran.r-project.org/web/packages/survminer/) was utilized for data visualization. After the hazard ratio (HR), 95% confidence intervals (95% CI) and log-rank P-values were computed, the Kaplan–Meier survival plots with visualized parameters were generated.

### Cell source

The human renal cell carcinoma cell lines ACHN and A498, and the human renal tubular epithelial cell line HK-2 were acquired from the Cell Bank of Type Culture Collection of Chinese Academy of Sciences and cultured in RPMI 1640 medium (Thermo Fisher Scientific, USA) with 10% fetal bovine serum (Gibco, USA). Both cell lines were maintained in a 5% CO_2_ incubator at 37 °C.

### Silencing of YBX3-siRNA

The YBX3 gene was used as a template to construct a lentiviral vector for small interfering RNA (siRNA) interference by Baioujing Medical Technology Co., Ltd. After having reached ~ 30% confluence, the cells were transfected with YBX3 siRNA lentivirus or control siRNA lentivirus and cultured for a further three days. The siRNA target sequences of YBX3 were as follows: siYBX3-1: 5’-CGGUUCAUCGAAAUCCAACUUTT-3’ (sense) and 5’-AAGUUG GAUU UCGAUGAACCGTT-3’ (antisense); siYBX3-2: 5’-CCGUCUGUUCGCCGUG GAUAUTT-3’ (sense) and 5’-AUAUCCACGGCGAACAGACGGTT-3’ (antisense); and siYBX3-3: 5’-GAGAUGGAGAAACUGUAGAGUTT-3’ (sense) and 5’-ACUCUACAGUUUCUCCAUCUCTT-3’ (antisense) and siCtrl: 5’- UUCUCCGAACGUGU-CACGUTT-3’. Finally, the transfection efficiency was detected using qRT-PCR and western blot analyses following the methods mentioned before (Emisoglu-Kulahli et al. 2021).

### Overexpression of YBX3

After the cells had reached ~ 30% confluence, they were transfected with the YBX3 gene plasmid or empty plasmid lentivirus particles obtained from Baioujing Medical Technology Co., Ltd., following the reagent manufacturer's instructions, and cultured for a further three days. Finally, the transfection efficiency was detected by qRT-PCR and western blot analyses. Bar graphs are the mean ± SD of three separate experiments.

### Western blot analysis

After total proteins had been extracted, 60 μg of the extracted protein was loaded per well on to the gel. The proteins were then separated by SDS-PAGE and transferred to a polyvinylidene fluoride membrane, and the membrane was blocked in 5% bovine serum albumin for 1 h. The primary anti-YBX3 antibody (cat. no. #ABS102405; 1:10,000 dilution, Absin Bioscience, Inc.) was incubated with the membrane overnight at 4 °C. The secondary goat anti-rabbit antibody (cat. no. # ab205718; 1:10,000, Abcam) was incubated at room temperature for 1 h. Subsequently, ECL working solution (Thermo Fisher Scientific, USA) was used for development of the bands, and the X-ray film was inserted into the cassette for fixing after exposure for 5–10 min. Finally, ImageJ software was used to analyze the optical density value of the bands. Each western blotting experiment was repeated three times.

### Cell proliferation assay

Cell Counting Kit-8 (CCK8) kit (Beyotime Biotechnology, China), assay was used to assess the proliferation of the cells following the previous method (Xu et al. 2023). In brief, 100 μl cell suspension was added to each well of five 96-well plates, and the cells were incubated at 37° for 24 h. Subsequently, 10 μl CCK8 solution was added on days 1, 2, 3, 4 and 5 and incubated for 2 h. Finally, the absorbance at 450 nm was measured using a microplate reader. Bar graphs are the mean ± SD of three separate experiments.

### Colony formation assay

The cells were inoculated in a 6-well plate (Thermo Fisher Scientific, USA) at a density of 1,000 cells/well. After culture for 2 weeks, the cells were fixed with 4% paraformaldehyde for 30 min at room temperature. The cells were then washed with phosphate-buffered solution (PBS) and stained with 0.1% crystal violet buffer for 20 min. Bar graphs are the mean ± SD of three separate experiments.

### Cell cycle assay

Cell cycle progression is one of the important indicators that is utilized to effectively reflect the proliferation of cells. cell-cycle analysis kit C1052 (Beyotime Biotechnology, China) was used in the present study to assess the cell cycle, and to investigate the proliferative abilities of cells. After the cell suspension was collected, the cells were washed with PBS, subsequently pre-cooled 75% ethanol was added in a vortex mixer, and the cells were fixed at 4 °C for 2 h. Finally, cells were stained with propidium iodide for 30 min in the dark. Bar graphs are the mean ± SD of three separate experiments.

### Scratch wound healing assay

After drawing two horizontal lines on the back of the 12-well plates (Thermo Fisher Scientific, USA), 1.5 × 10^5^ cells either silenced for the YBX3 gene or overexpressing the YBX3 gene were added, according to the experimental design groups. When the cells had reached a confluence of > 90% the next day, the scratch was made with a 10 µl pipette tip perpendicular to the horizontal line, and the medium was exchanged for a low-concentration serum medium. Finally, images were captured at 0, 24 and 48 h. Bar graphs are the mean ± SD of three separate experiments.

### Cell invasion assay

For the Transwell assays, the cell suspension was prepared at a final density of 6 × 10^4^ cells/well with serum-free medium. After the Matrigel™ matrix (Corning, USA) layer had been rehydrated, 100 µl serum-free cell suspension was added to the upper chamber, and 600 µl medium containing 10% fetal bovine serum was added to the lower chamber; the cells were then incubated at 37 °C for 24 h. The non-transferred cells were gently removed with a cotton swab, and then the plate was washed 3 times with PBS. Finally, each chamber was stained with 0.1% crystal violet after fixing with 4% paraformaldehyde. Six fields of view were randomly selected from each membrane, and the cells were counted under a microscope. Bar graphs are the mean ± SD of three separate experiments.

### Apoptosis assay

The cell suspension was prepared at a final density of 6 × 10^4^ cells/well. After washing with PBS that had been pre-cooled at 4 °C, 2 μl Annexin V-APC and PI (Thermo Fisher Scientific, USA) were added for staining for 20 min in dark, and then the extent of apoptosis was assessed by flow cytometric analysis (MilliporeSigma, USA). These experiments were performed in triplicate. Bar graphs are the mean ± SD of three separate experiments.

### Statistical analysis

Wilcoxon rank sum test, Spearman’s correlation test and the Kruskal–Wallis test was employed for bioinformatics analysis, as described above. For the YBX3 expression analysis in TCGA-KIRC dataset, paired Student’s t-test was employed in paired samples, while unpaired Student’s t-test was employed for other data. Three biological replicates were performed in all *in-vitro* experiments and data were statistically analyzed using a two-tailed Student's t test for siRNA selection, two-way ANOVA for cell cycle, and repeated-measures ANOVA for cell proliferation and scratch wound healing assays. All analyses were performed using GraphPad Prism 6 software (GraphPad Software, Inc.), and *P* < 0.05 was considered to indicate a statistically significant value.

## Results

### Abnormally high expression of YBX3 in ccRCC

**Fig. 1:**
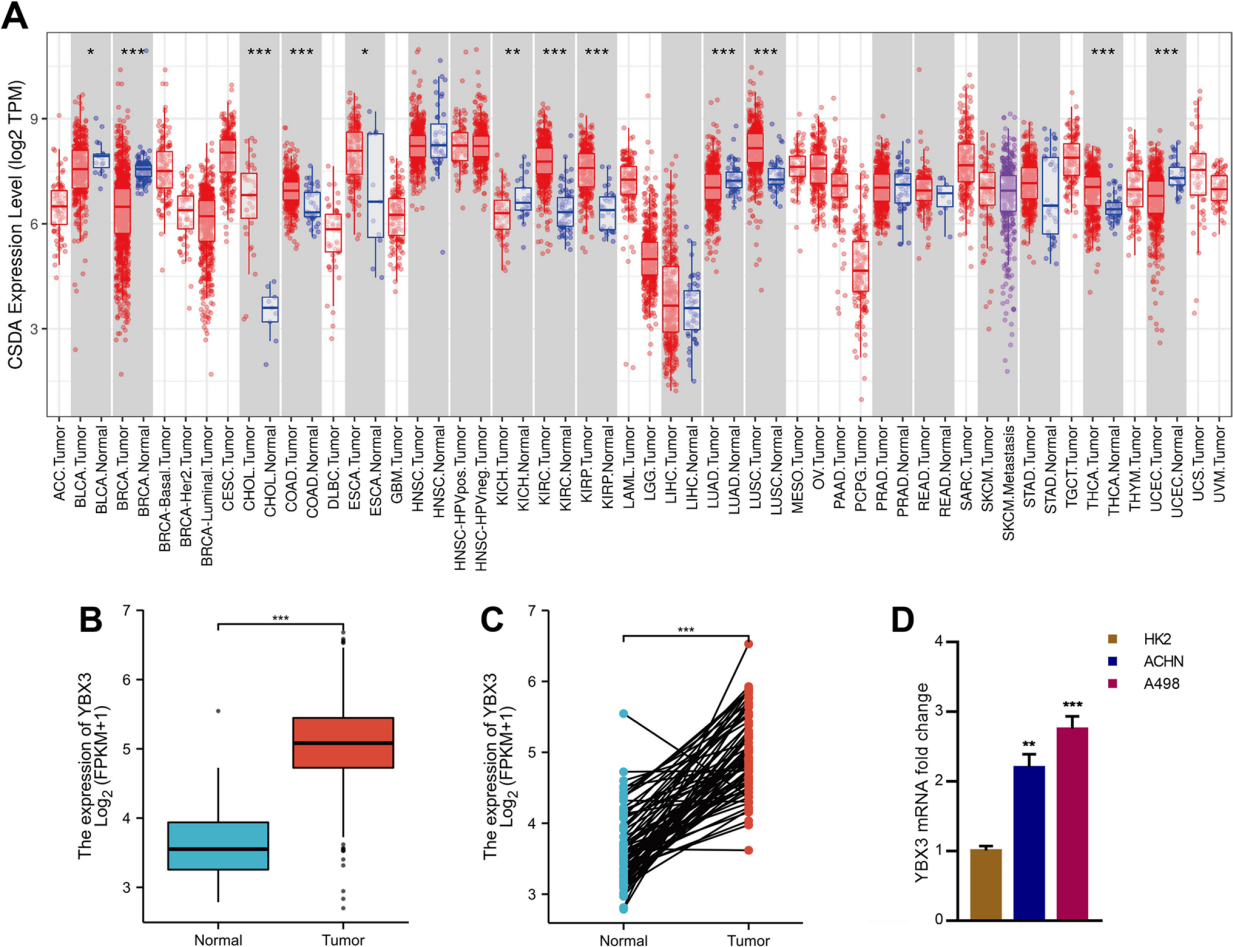
YBX3 is abnormally high expressed in ccRCC. (**A**) The expression level of YBX3 of pan cancers in the TCGA database was analyzed via Timer 2.0 tool. Blue marks normal tissue, red marks tumor tissue, and purple marks metastatic tumor tissue. (**B**) YBX3 expression levels in ccRCC and paracancerous samples. (**C**)YBX3 expression levels in ccRCC paired samples. (**D**) The mRNA level of YBX3 was detected by qPCR. *P < 0.05. **P < 0.01.***P < 0.001

First, YBX3 was found to be significantly expressed in the various types of cancer tissues analyzed, namely cholangiocarcinoma (CHOL) (*P* < 0.001), colon adenocarcinoma (COAD) (*P* < 0.05), esophageal carcinoma (ESCA) (*P* < 0.001), ccRCC (*P* < 0.001), renal papillary cell carcinoma (KIRP) (*P* < 0.001), lung squamous cell carcinoma (LUSC) (*P* < 0.001) and thyroid carcinoma (THCA) (*P* < 0.001) (Fig. 1A). Secondly, the expression of YBX3 was found to be markedly higher in ccRCC specimens (n = 541, P = 4.27e-33, 95% CI: 1.4476–1.777) compared with the paracancerous samples (n = 72) in TCGA KIRC dataset (Fig. 1B). Additionally, compared with the matched paracancerous samples (n = 72), a significantly high expression level of YBX3 was identified in ccRCC samples (n = 72, P = 4.28e-23, 95% CI: 1.3737–1.8079) (Fig. 1C). Through qRT-PCR analysis of the cancer cells, subsequent experiments verified that YBX3 was highly expressed in the A498 cell line, whereas the lowest levels of expression occurred in ACHN cells (Fig. 1D).

### Correlation between YBX3 expression and immune infiltration

**Fig. 2:**
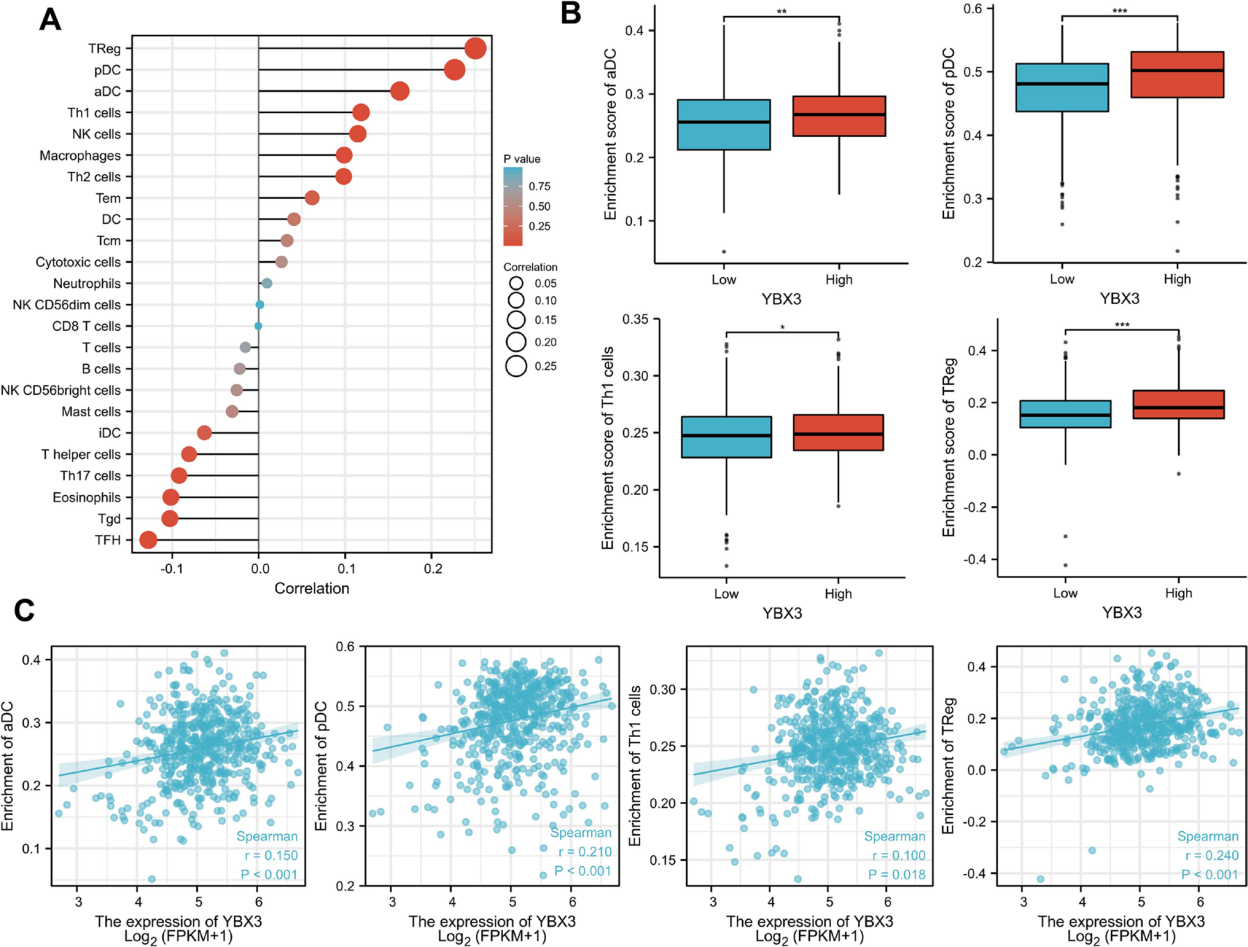
The YBX3 expression was correlation with the immune infiltration. (**A**) Lollipop plot of the correlation of YBX3 expression with immune infiltration. Spearman correlation was employed. (**B**) Boxplot of differences in immune cell infiltration levels between YBX3 high and low groups. (**C**) Scatter and correlograms of immune cell infiltration and YBX3 expression. *P < 0.05, **P < 0.01, ***P < 0.001

The data results from the lollipop plot indicated a positive correlation between the expression of YBX3 and the abundance of immune cells such as aDC, pDC, Th1, and Treg cells, while a negative correlation was observed with the abundance of immune cells such as TFH, Tgd, eosinophils, and Th17 cells (Fig. 2A, Table S1). Notably, a strong correlation was found between the infiltration level of immune cells and the expression of YBX3, prompting subsequent box plot and scatterplot analyses to demonstrate the relationship between the expression of YBX3 and the immune infiltration level of aDC, pDC, Th1, and TReg cells (Fig. 2B and C). These findings suggested that YBX3 may regulate the immune microenvironment through the aforementioned immune cells, thereby modulating tumor progression.

In order to analyze the clinicopathological variables, a total of 265 ccRCC samples with low YBX3 expression data, as well as 265 ccRCC samples with high YBX3 expression data, were analyzed (Table 1). The Shapiro–Wilk normality test results showed that the samples did not meet the normality test threshold (*P* < 0.05), and so the Wilcoxon rank sum test was further employed (Table S2).

**Table 1:** Correlation between YBX3 expression and clinicopathological features of ccRCC

Characteristic	levels	Low expression of YBX3	High expression of YBX3	*p*
*n*		265	265	
T stage, *n* (%)	T1	157 (29.6%)	114 (21.5%)	< 0.001
	T2	38 (7.2%)	31 (5.8%)	
	T3	67 (12.6%)	112 (21.1%)	
	T4	3 (0.6%)	8 (1.5%)	
N stage, *n* (%)	N0	128 (50.2%)	111 (43.5%)	0.616
	N1	7 (2.7%)	9 (3.5%)	
M stage, *n* (%)	M0	219 (44%)	201 (40.4%)	0.093
	M1	32 (6.4%)	46 (9.2%)	
Pathologic stage, *n* (%)	Stage I	152 (28.8%)	113 (21.4%)	< 0.001
	Stage II	34 (6.5%)	23 (4.4%)	
	Stage III	46 (8.7%)	77 (14.6%)	
	Stage IV	31 (5.9%)	51 (9.7%)	
Primary therapy outcome, *n* (%)	PD	2 (1.4%)	9 (6.5%)	0.027
	SD	2 (1.4%)	3 (2.2%)	
	PR	2 (1.4%)	0 (0%)	
	CR	68 (49.3%)	52 (37.7%)	
Gender, *n* (%)	Female	112 (21.1%)	74 (14%)	< 0.001
	Male	153 (28.9%)	191 (36%)	
Race, *n* (%)	Asian	7 (1.3%)	1 (0.2%)	0.069
	Black or African American	31 (5.9%)	25 (4.8%)	
	White	225 (43%)	234 (44.7%)	
Age, *n* (%)	< = 60	134 (25.3%)	130 (24.5%)	0.794
	> 60	131 (24.7%)	135 (25.5%)	
Histologic grade, *n* (%)	G1	10 (1.9%)	4 (0.8%)	0.200
	G2	117 (22.4%)	110 (21.1%)	
	G3	99 (19%)	107 (20.5%)	
	G4	32 (6.1%)	43 (8.2%)	
Serum calcium, *n* (%)	Elevated	3 (0.8%)	7 (1.9%)	0.400
	Low	100 (27.5%)	103 (28.4%)	
	Normal	67 (18.5%)	83 (22.9%)	
Hemoglobin, *n* (%)	Elevated	1 (0.2%)	4 (0.9%)	0.031
	Low	120 (26.7%)	141 (31.3%)	
	Normal	104 (23.1%)	80 (17.8%)	
Laterality, *n* (%)	Left	119 (22.5%)	130 (24.6%)	0.407
	Right	145 (27.4%)	135 (25.5%)	
OS event, *n* (%)	Alive	198 (37.4%)	159 (30%)	< 0.001
	Dead	67 (12.6%)	106 (20%)	
PFI event, *n* (%)	Alive	207 (39.1%)	163 (30.8%)	< 0.001
	Dead	58 (10.9%)	102 (19.2%)	
DSS event, *n* (%)	Alive	223 (43%)	188 (36.2%)	< 0.001
	Dead	38 (7.3%)	70 (13.5%)	
Age, mean ± SD		60.14 ± 12.09	60.99 ± 12.2	0.417

(Progressive disease); SD (stable disease); PR (partial response); CR (Compete response); G1 (Grade 1); G2 (Grade 2); G3 (Grade 3); G4 (Grade 4); OS (Overall survival); PFI (Progression Interval); DSS (Disease Free Survival)

**Fig. 3:**
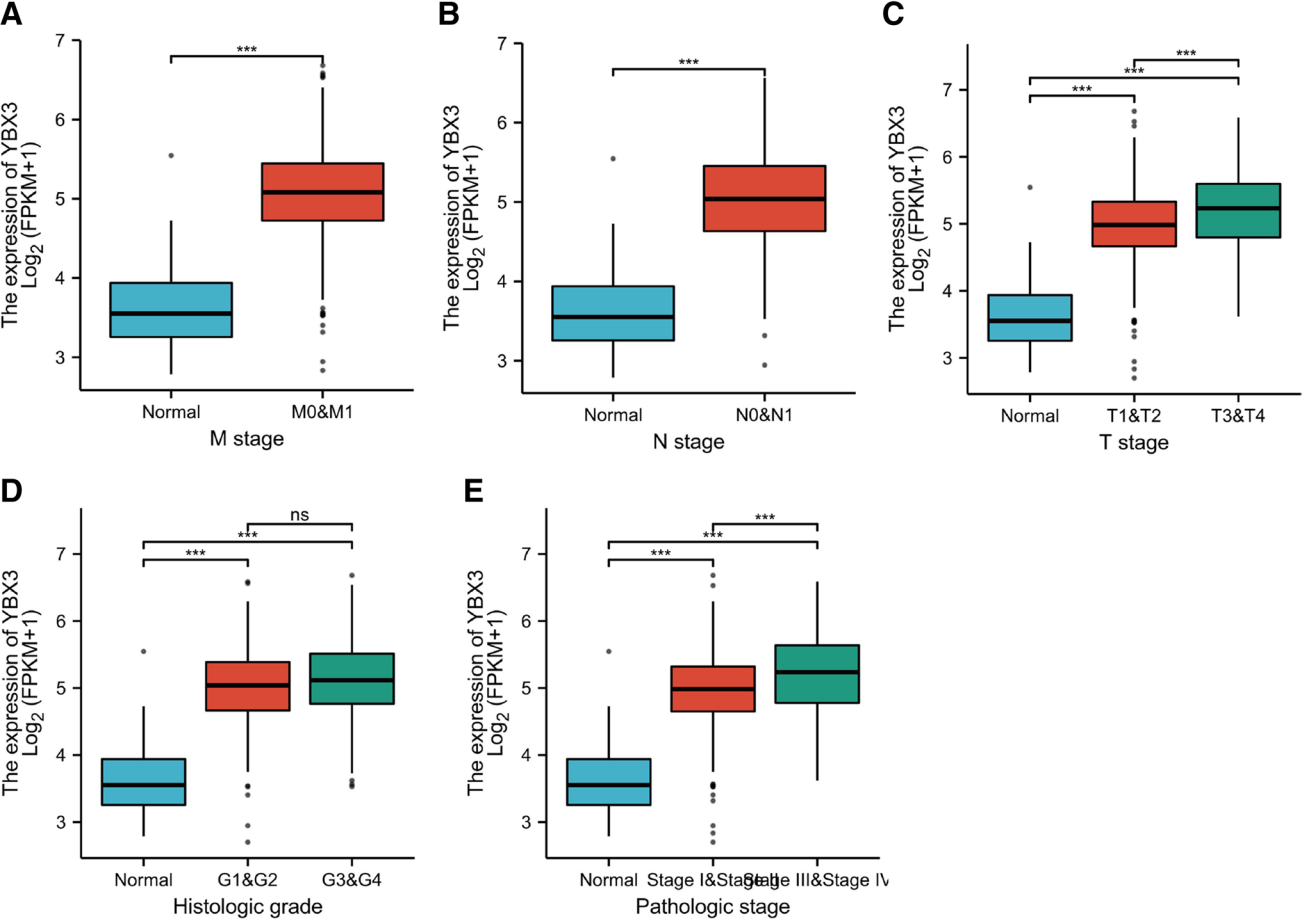
Differential YBX3 expression related to clinicopathological characteristics in ccRCC patients of TCGA cohort. (**A**) The difference of YBX3 expression in samples with different M stages. (**B**)The difference of YBX3 expression in samples with different N stages. (**C**)The difference of YBX3 expression in samples with different T stages. (**D**) The difference of YBX3 expression in samples with different histological grades. (**E**) The difference of YBX3 expression in samples with different pathologic stages. ***P < 0.001. ns: no significance

In the cohort exhibiting low YBX3 expression data, comprising of 153 males and 112 females, the mean age was 60.14 years (range: 48–73 years). Conversely, in the cohort with high YBX3 expression data, consisting of 191 males and 74 females, the mean age was 60.99 years (range: 48–73 years). As depicted in Fig. 3A-C, no significant correlation was established between overexpressed YBX3 and tumor histological grade (grade 3 & 4 vs. grade 1 & 2; *P* > 0.05). However, significant correlations were observed between overexpressed YBX3 and the cancer stage (stage III and IV vs. stage I and II; *P* < 0.001), T stage (T3 and 4 vs. T1 and 2; *P* < 0.001), sex (female vs. male; *P* < 0.001), primary therapy outcome [progressive disease (PD) and stable disease (SD) and partial response (PR) vs. complete response (CR); *P* < 0.05], hemoglobin (elevated vs. low and normal; *P* < 0.05), and OS [progression free interval (PFI) and disease specific survival (DSS) events (alive vs. dead); *P* < 0.001] (Table 2). Increased YBX3 expression levels in ccRCC were positively associated with T stage [odds ratio (OR) = 1.926], pathological stage (OR = 1.830) and hemoglobin (OR = 1.558 for low and elevated vs. normal). In contrast, increased YBX3 expression levels in ccRCC were significantly (all *P* < 0.05) negatively associated with primary therapy outcome (OR = 0.170 for SD and PR and CR vs. PD). The results suggested that ccRCC with overexpression of YBX3 had a poorer prognosis, was associated with more male patients, and had an elevated rate of tumor progression to the advanced stages compared to ccRCC with low YBX3 expression.

**Table 2:** Univariate regression analysis and multivariate analysis of prognostic variables in ccRCC patients

Characteristics	Total (N)	Odds Ratio (OR)	*P* value
T stage (T2&T3&T4 vs. T1)	530	1.926 (1.365–2.724)	< 0.001
N stage (N1 vs. N0)	255	1.483 (0.535–4.272)	0.449
M stage (M1 vs. M0)	498	1.566 (0.963–2.574)	0.073
Pathologic stage (Stage II&Stage III&Stage IV vs. Stage I)	527	1.830 (1.297–2.589)	< 0.001
Primary therapy outcome (SD&PR&CR vs. PD)	138	0.170 (0.025–0.691)	0.027
Histologic grade (G2&G3&G4 vs. G1)	522	2.621 (0.864–9.654)	0.107
Gender (Male vs. Female)	530	1.889 (1.317–2.722)	< 0.001
Race (Black or African American&White vs. Asian)	523	7.082 (1.248–132.866)	0.068
Age (> 60 vs. < = 60)	530	1.062 (0.756–1.494)	0.728
Serum calcium (Elevated&Low vs. Normal)	363	0.862 (0.566–1.311)	0.488
Hemoglobin (Low&Elevated vs. Normal)	450	1.558 (1.068–2.278)	0.022
Laterality (Right vs. Left)	529	0.852 (0.605–1.199)	0.359

(Progressive disease); SD (stable disease); PR (partial response); CR (Compete response); G1 (Grade 1); G2 (Grade 2); G3 (Grade 3); G4 (Grade 4)

### Survival prognosis analysis

**Fig. 4:**
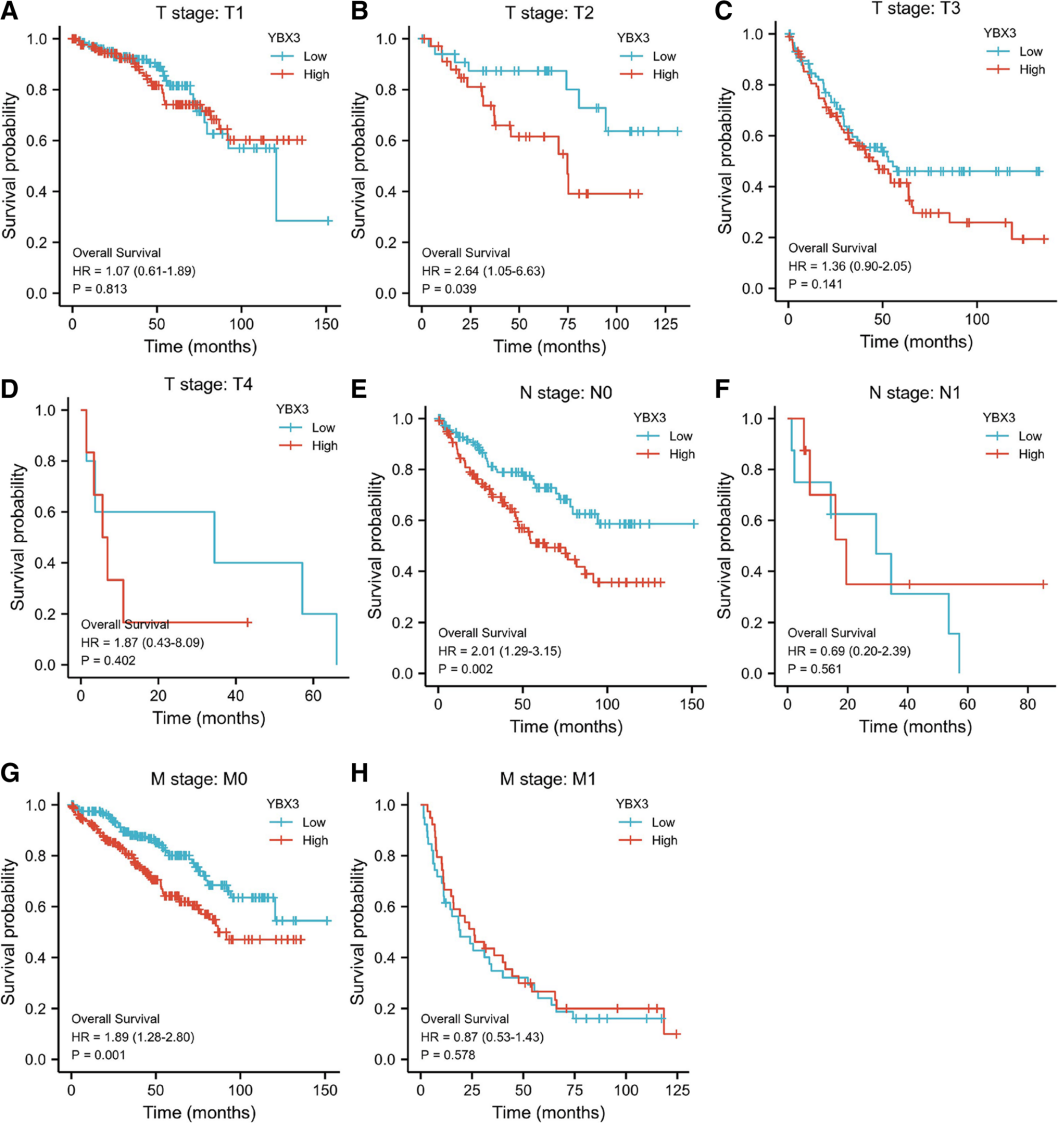
TNM stages subgroup Kaplan–Meier analysis between YBX3 high and low groups in ccRCC. (**A**) Kaplan–Meier curves for overall survival of T1 subgroups for TCGA-KIRC samples. (**B**) Kaplan–Meier curves for overall survival of T2 subgroups for TCGA-KIRC samples. (**C**) Kaplan–Meier curves for overall survival of T3 subgroups for TCGA-KIRC samples. (**D**) Kaplan–Meier curves for overall survival of T4 subgroups for TCGA-KIRC samples. (**E**) Kaplan–Meier curves for overall survival of N0 subgroups for TCGA-KIRC samples. (**F**) Kaplan–Meier curves for overall survival of N1 subgroups for TCGA-KIRC samples. (**G**) Kaplan–Meier curves for overall survival of M0 subgroups for TCGA-KIRC samples. (**H**) Kaplan–Meier curves for overall survival of M1 subgroups for TCGA-KIRC samples

Looking at the TNM stages, the results of subgroup survival analyses of the OS rates revealed that patients’ prognosis with high expression of YBX3 (“YBX3-high”) was poorer in the T2 (HR: 2.64; 95% CI: 1.05–6.63; *P* < 0.05), M0 (HR: 1.89; 95% CI: 1.28–2.80; *P* < 0.01) and N0 (HR: 2.01; 95% CI: 1.29–3.15; *P* < 0.001) subgroups (Fig. 4). This indicated that YBX3 was associated with an unfavorable prognosis in patients with advanced ccRCC, or patients with no distant metastasis and lymphatic metastasis. However, no meaningful differences in survival were identified among the T1 (HR: 1.07; 95% CI: 0.61–1.89; P = 0.813), T3 (HR: 1.36; 95% CI: 0.90–2.05; P = 0.141), T4 (HR: 1.87; 95% CI: 0.43–8.09; P = 0.402), M1 (HR: 0.87; 95% CI: 0.53–1.43; P = 0.578) and N1 (HR: 0.69; 95% CI: 0.20–2.39; P = 0.561) subgroups of OS.

### Silencing YBX3 inhibits renal cancer progression in vitro

**Fig. 5:**
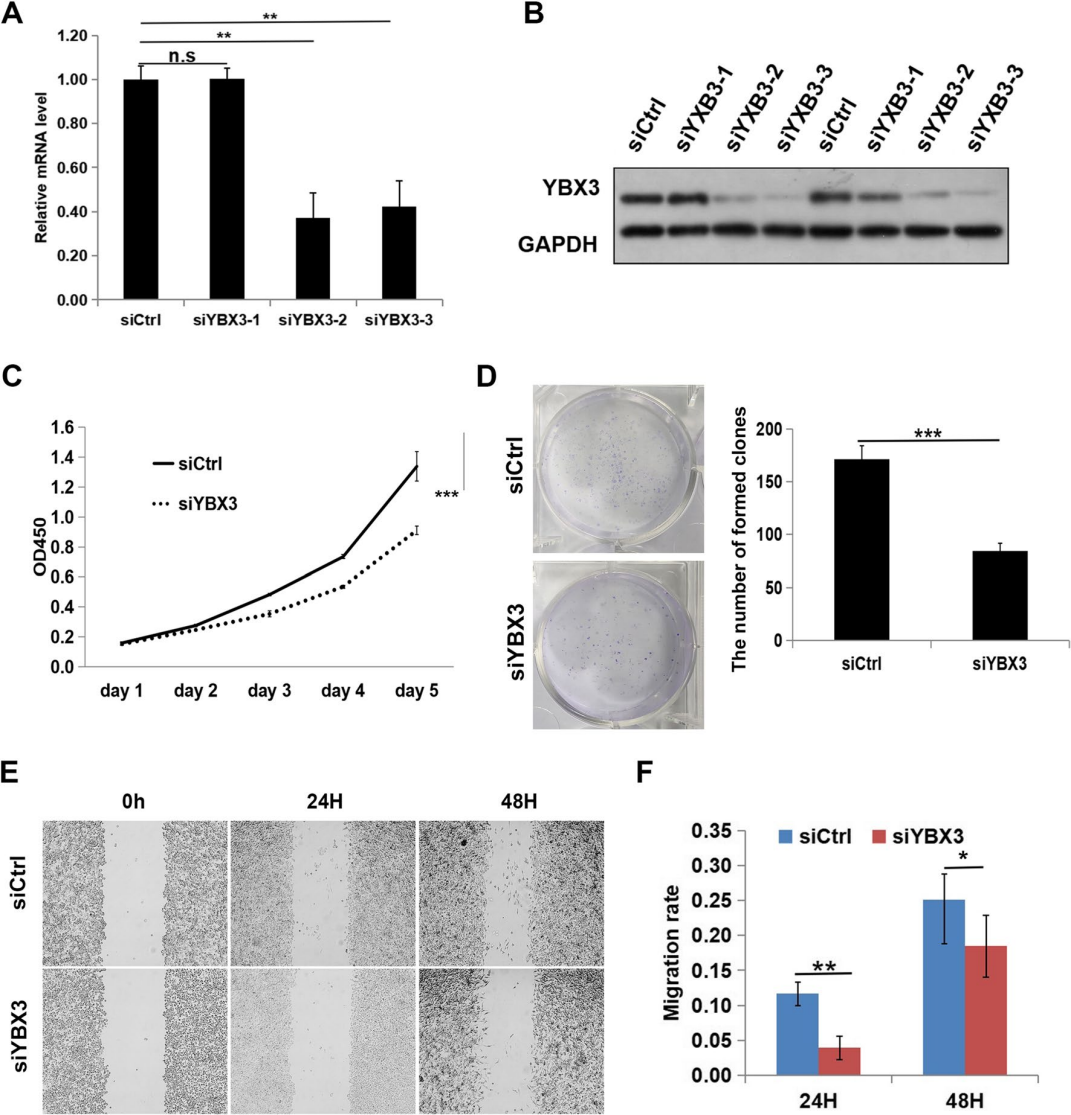
Silencing YBX3 inhibits renal cancer proliferation and migration in vitro. (**A**) The YBX3 mRNA expression were examined through qRT-PCR. (B) The protein level of YBX3 were examined through western blot. (**C**) Proliferation of A498 cells was investigated via CCK8 assays. (**D**) Clone formation ability of A498 cells was investigated through clone formation assay. (**E** and **F**) Migration of A498 cells were discovered by way of scratch healing assay. Data were expressed as the mean ± s.d., At least three individual experiments were performed in each treatment group. **P < 0.01, ***P < 0.001. ns: no significance. Three biological replicates were performed and the result was subjected to statistical analysis

**Fig. 6:**
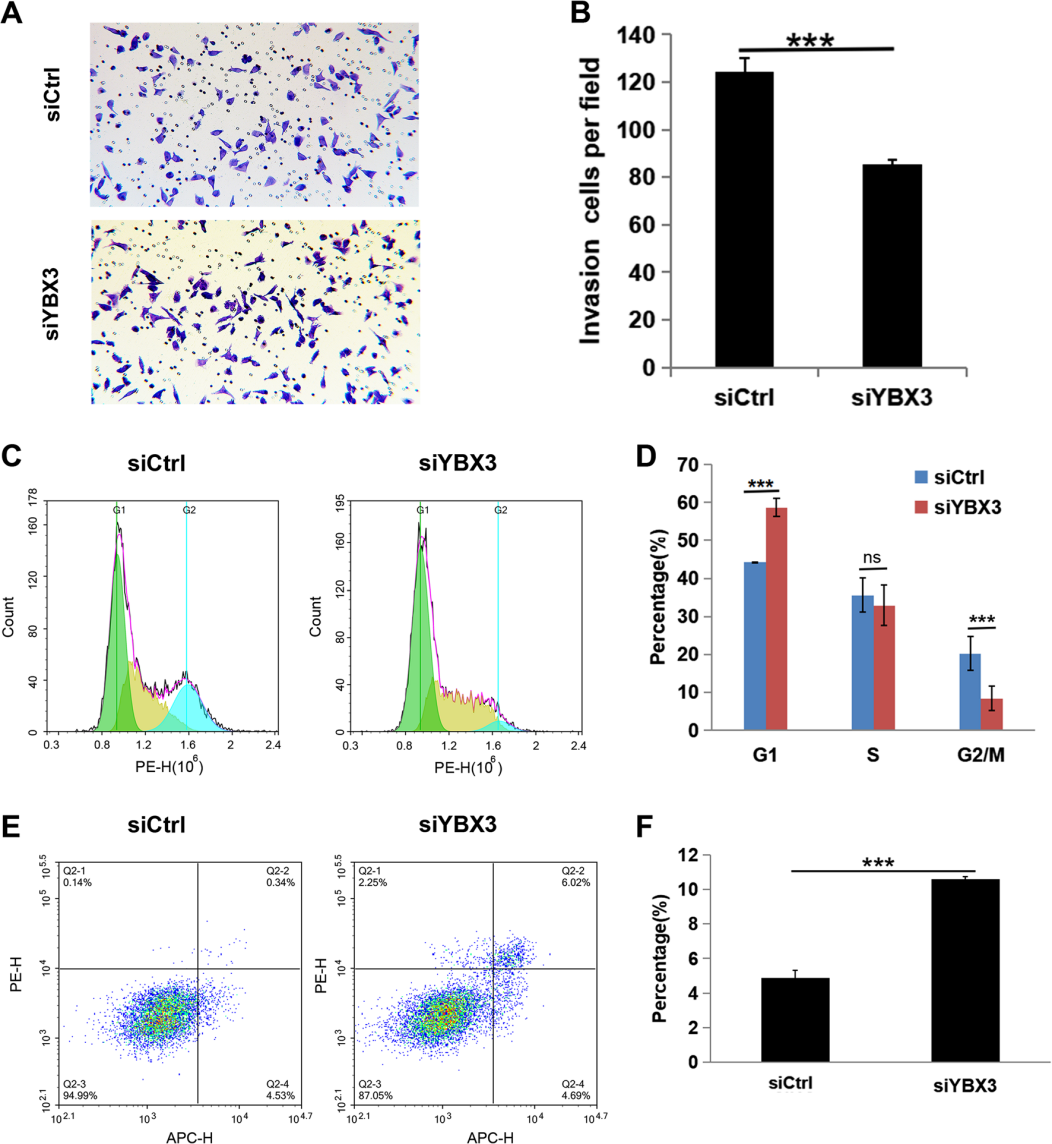
Silencing YBX3 inhibits renal cancer invasion and induces cell cycle arrest and apoptosis in vitro. (**A** and **B**) Invasion of A498 cells were discovered by way of Transwell assay. (**C** and **D**) The cell cycle of A498 was detected via flow cytometry. (**E** and **F**) The proportion of apoptotic cells in A498 cell line was assessed by flow cytometry. Data were expressed as the mean ± s.d., At least three individual experiments were performed in each treatment group. **P < 0.01, ***P < 0.001. ns: no significance. Three biological replicates were performed and the result was subjected to statistical analysis

The ability to proliferate, migrate, invade tissues, and evade apoptosis are important characteristics of tumor cells in terms of the progression of malignancies. In order to clarify the effect of YBX3 on the progression of ccRCC in vitro, siRNAs were first used to knock down YBX3 in the ccRCC cell line A498, which highly expresses YBX3, and the efficiency of the knockdown was confirmed by qRT-PCR and western blot analyses. As shown in Fig. 5A and B, sequence-2 and -3 were able to effectively inhibit the expression of YBX3 (*P* < 0.001). Combined with the expression data of RNA and protein after transfections of 3 siRNA fragments, the siYBX3-3 was employed for subsequent cytology experiments. The effect of YBX3 knockdown on the proliferation of A498 cell line was examined by a CCK-8 proliferation assay and a clone formation assay. As shown in Fig. 5C-D, consistently, siYBX3-3 group cells showed significantly decreased proliferation and clone formation, ***P < 0.001. The migration and of different cell lines were evaluated via the scratch assay, and quantitative analysis of the test showed that siCtrl group cells migrated towards the scratch better than normal siYBX3-3 group cells (Fig. 5E-F, ***P < 0.001). Subsequently, cell migration ability was detected via transwell assay. To identify the function of YBX3 in migration, we used transwell assay to detect the cell migration ability. The result was consistent with scratch assay experiments that the numbers of cells that traversed the Transwell were significantly reduced by YBX3 knockdown in the A498 cells (Fig. 6A-B, ***P < 0.001). The cell cycle analysis showed that knockdown of YBX3 interrupted cell cycle progression. YBX silencing reduced the proportion of A549 cells in G2/M phases and increased cells in G1 phase, suggesting cell cycle arrest at either G1 phase (Fig. 6C-D, ***P < 0.001). Moreover, the level of apoptosis was significantly increased after YBX3 knockdown in A498 cells (*P* < 0.001) (Fig. 6E-F). Taken together, the above results comprehensively demonstrated that inhibition of YBX3 could restrain the progression of ccRCC in vitro.

### Overexpression of YBX3 promotes renal cancer progression in vitro

**Fig. 7:**
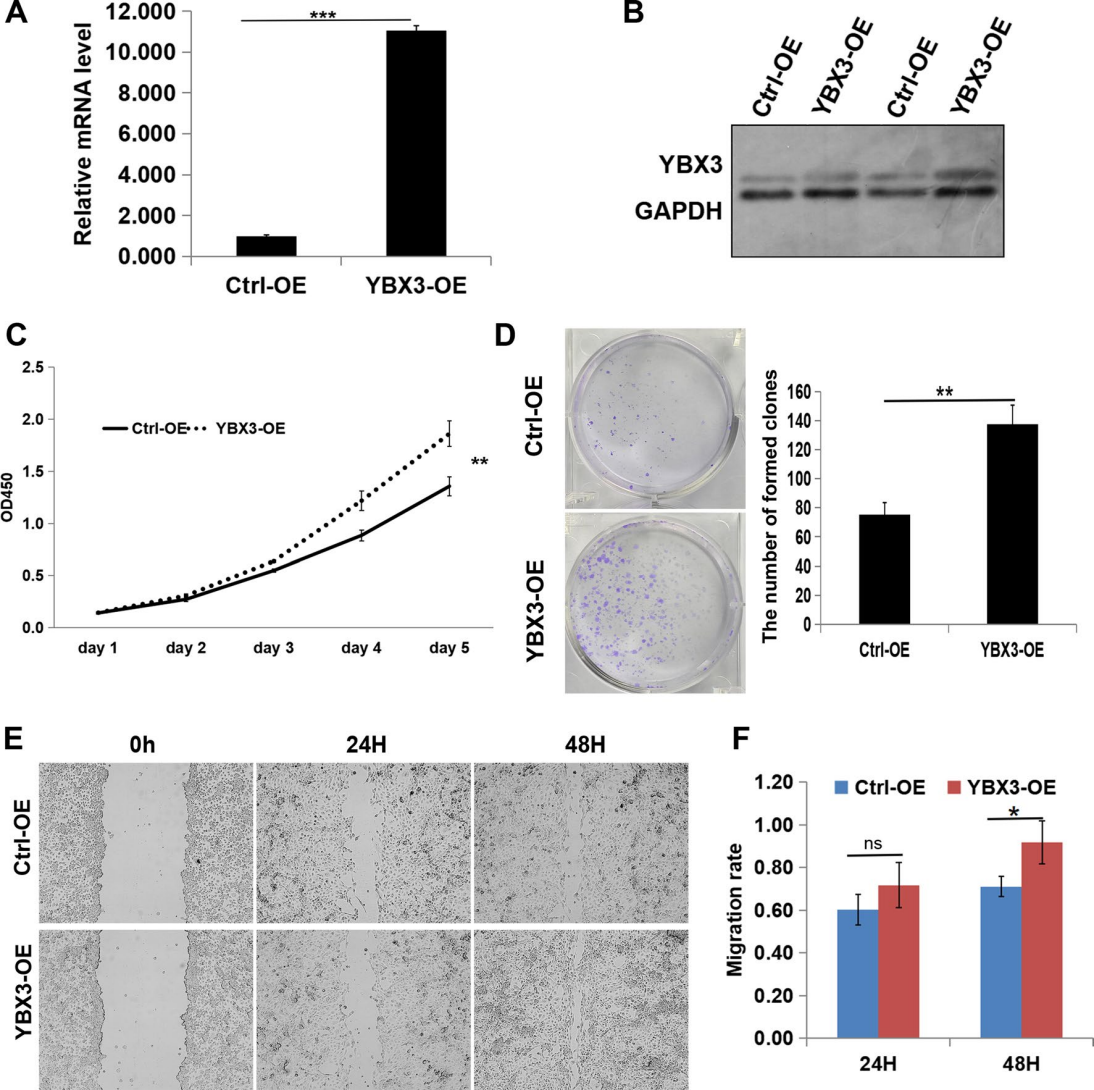
Overexpression of YBX3 promotes renal cancer proliferation and migration in vitro. (**A**) The YBX3 mRNA expression were examined through qRT-PCR. (**B**) The protein level of YBX3 were examined through western blot. (**C**) Proliferation of ACHN cells was investigated via CCK8. (**D**) Clone formation ability of ACHN cells was investigated via clone formation assay. (**E** and **F**) Migration of ACHN cells were discovered by way of scratch healing assay. Data were expressed as the mean ± s.d., At least three individual experiments were performed in each treatment group. *P < 0.05, **P < 0.01, ***P < 0.001. ns: no significance. Three biological replicates were performed and the result was subjected to statistical analysis

**Fig. 8:**
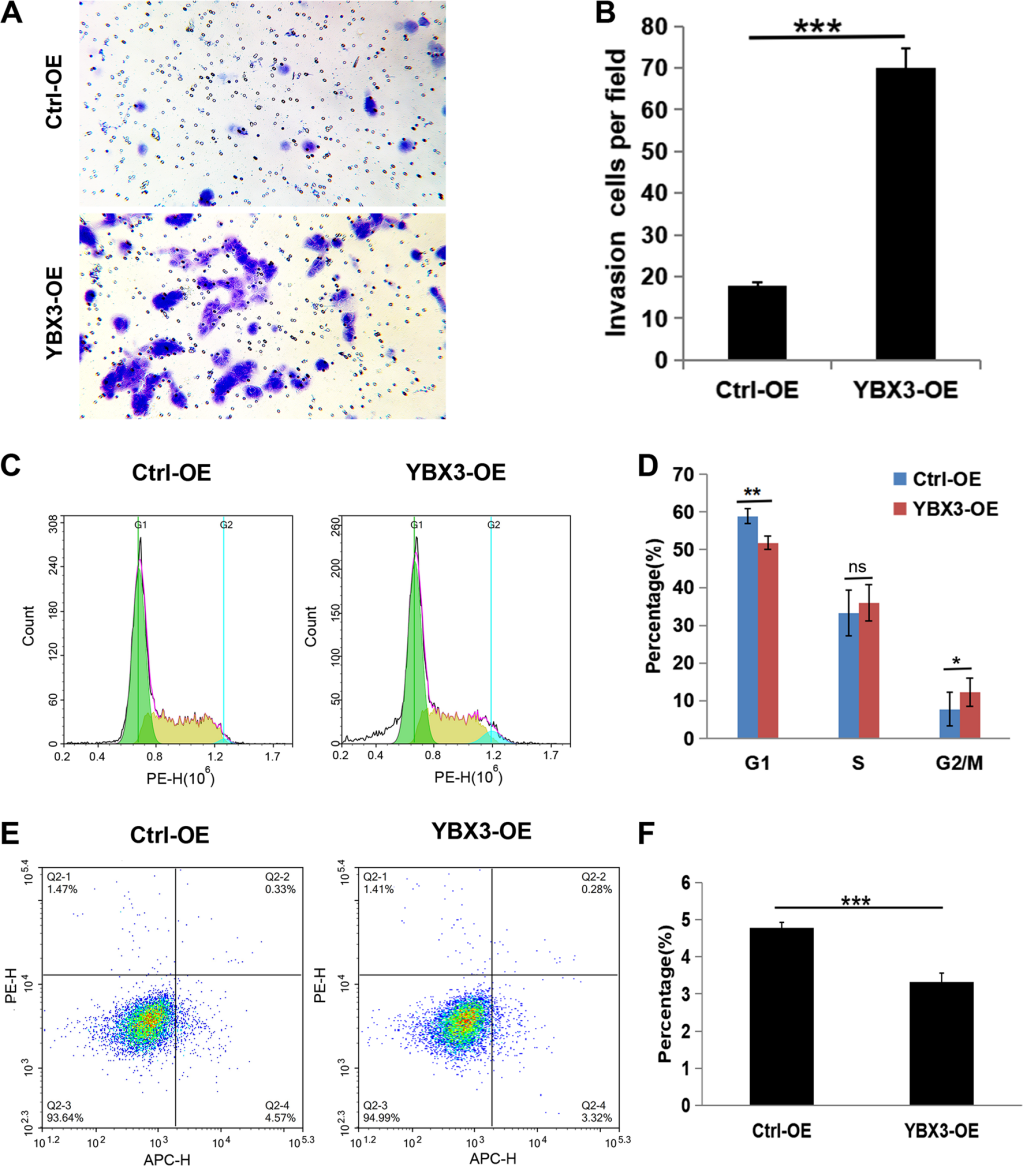
Overexpression of YBX3 promotes renal cancer invasion and inhibits cell cycle arrest and apoptosis in vitro. (**A** and **B**) Invasion of ACHN cells were discovered by transwell assay. (**C** and **D**) The cell cycle of ACHN was detected via flow cytometry. (**E** and **F**) The proportion of apoptotic cells in ACHN cell line was assessed by flow cytometry. Data were expressed as the mean ± s.d., At least three individual experiments were performed in each treatment group. *P < 0.05, **P < 0.01, ***P < 0.001. ns: no significance. Three biological replicates were performed and the result was subjected to statistical analysis

To further elucidate the effect of YBX3 on the progression of ccRCC in vitro, YBX3 was overexpressed in the ACHN cell line, which expressed only a low level of YBX3, and the overexpression was confirmed via qRT-PCR and western blot analyses (Fig. 7A-B, *P* < 0.001). The overexpression plasmid successfully increased the expression level of YBX3 in the cells. Subsequently, cell proliferation, migration, invasion, and apoptosis detection experiments were conducted, revealing that overexpressed YBX3 effectively promoted the proliferation (*P* < 0.01), clone formation ability (*P* < 0.01), cell cycle progression (*P* < 0.01), migration (*P* < 0.05), and invasion (*P* < 0.001) of the ACHN cells, while significantly inhibiting apoptosis (*P* < 0.001) (Fig. 7–8). Collectively, these results comprehensively demonstrated that overexpression of YBX3 could facilitate the progression of ccRCC in vitro.

## Discussion

Renal carcinoma, a highly malignant tumor (Motzer et al. 2020), is frequently associated with ccRCC, the most common pathological subtype (Henriksen and Chang 2020). Patients with ccRCC often experienced high rates of metastasis and mortality, and had a dismal prognosis (Li et al. 2021). Targeted therapies aimed at different molecular characteristics of ccRCC have been implemented, but their efficacy was limited. Therefore, a systematic analysis of ccRCC gene characteristics was essential to identify novel biomarkers and prognostic predictors.

YBX3, a regulator of amino acid transporters and essential for cell growth and proliferation (Wang et al. 2020), has been shown to be upregulated in various tumor cells and to participate in tumor progression (Fan et al. 2021). In this study, we conducted bioinformatics analysis based on TCGA database and found that YBX3 was highly expressed in ccRCC tissues. High expression levels of YBX3 in ccRCC were associated with more advanced stages, poor prognosis, and a higher proportion of male patients (Sun et al. 2022) have indicated that YBX3 was highly expressed in cholangiocarcinoma, colon carcinoma, renal clear cell carcinoma, renal papillary cell carcinoma, lung squamous cell carcinoma, and thyroid carcinoma. Furthermore, it was associated with poor prognosis of lung squamous cell carcinoma and ccRCC. Moreover, the expression of YBX3 was positively correlated with the infiltration of multiple immune cells. In breast and ovarian cancer cohorts, higher YBX3 expression was significantly associated with chemotherapy resistance. Our study has found an overall significant difference (*P* < 0.001) in YBX3 expression in ccRCC compared with normal tissues. Although YBX3 was highly expressed in low-grade tumors and less expressed in high-grade tumors according to tumor grade, no significant differences were identified. Additionally, bioinformatics analysis has shown that these RNA transcripts were associated with clear individual variations and heterogeneity and, in terms of identification purposes, YBX3 may potentially serve as a moderate molecular marker for ccRCC tissues, which was consistent with previous reports. An elevated expression of YBX3 in gastric cancer has been linked to advanced clinicopathological features, shorter survival time, and poor prognosis, which was consistent with the findings of Xiang and colleagues (Xiang et al. 2020).

Immunotherapy has become a powerful clinical strategy for treating cancer patients, particularly those with metastatic spread (Kennedy and Salama 2020; Yu et al. 2022; Zheng et al. 2023). There has been significant progress in the treatment of patients with ccRCC, with improved knowledge of disease biology and the introduction of targeted agents and immunotherapies (Atkins and Tannir 2018). Our study has shown that YBX3 was also upregulated in ccRCC and was positively correlated with the infiltration of Treg cells, DC cells, and Th1 cells. The high expression of YBX3 in tumors may be associated with the increased infiltration of these immune cells. Tanaka and Sakaguchi (Tanaka and Sakaguchi 2019) have suggested that Treg-cell mediated suppression of anti-tumor immune responses occurred in cancer tissues. However, this study did not explain the relationship between YBX3 expression and Treg-cells. The present study has identified a positive association between the expression level of YBX3 and the abundance of immune cells such as aDC, pDC, Th1, and Treg cells, suggesting that it would be advantageous to develop treatment strategies that enhance the cancer immunity effect by increasing the infiltration of T cells into tumor tissues (Dora et al. 2021) have also found a close correlation between YBX3 and the tumor immune response. Statistics on YBX3 pan cancer expression, immune infiltration, and survival prognosis were obtained using various bioinformatics-based online analysis tools. However, the limited and scant experimental data obtained in the present study cannot serve as a reliable basis for prognosis and therapeutic options. Therefore, more comprehensive, and enriched studies are urgently required.

In this study, the association between YBX3 and the progression of ccRCC was confirmed by analyzing the proliferation, migration, invasion, extent of apoptosis, and other biological behaviors of cells in vitro after both silencing and overexpressing the YBX3 gene. Several previous studies have also demonstrated that YBX3 overexpression promotes cell proliferation and migration while decreasing the level of apoptosis (Di Mauro et al. 2020; Zhang et al. 2017). However, the underlying mechanism of how the YBX3 gene promotes the development of ccRCC remains unclear and requires further exploration.

## Conclusion

In conclusion, to the best of our knowledge, the present study has reported for the first time on the association of YBX3 with ccRCC progression and prognosis in vitro through bioinformatics and cell biological function analyses. YBX3 may be an effective ccRCC therapeutic target or a biomarker for prognosis prediction, and therefore this study has provided a valuable reference for clinical practice.

## Supplementary information

Below is the link to the electronic supplementary material.

Supplementary file1 (DOCX 17 KB)

Supplementary file2 (DOCX 19 KB)

## Data Availability

The dataset used and/or analyzed in this study is available from the corresponding author on reasonable request.
