# Supplementary material for: Identification of RNA-binding protein YBX3 as an oncogene in clear cell renal cell carcinoma
Source: Funct Integr Genomics. 2023 Jul 7;23(3):225. doi: 10.1007/s10142-023-01145-6 (PMC10329074; doi:10.1007/s10142-023-01145-6)
Supplement: Supplementary file 1 — Supplementary file1 (DOCX 17 KB) [file 10142_2023_1145_MOESM1_ESM.docx]

Table S1 Correlation coefficients between YBX3 expression and immune cell infiltration.

| variable | Cells | Correlation coefficient (Spearman) | P value (Spearman) |
| --- | --- | --- | --- |
| YBX3 | TFH | -0.146 | <0.001 |
| YBX3 | aDC | 0.151 | <0.001 |
| YBX3 | pDC | 0.213 | <0.001 |
| YBX3 | TReg | 0.236 | <0.001 |
| YBX3 | Tgd | -0.118 | 0.006 |
| YBX3 | Eosinophils | -0.115 | 0.008 |
| YBX3 | Th1 cells | 0.103 | 0.018 |
| YBX3 | Macrophages | 0.102 | 0.019 |
| YBX3 | Th2 cells | 0.097 | 0.026 |
| YBX3 | Th17 cells | -0.094 | 0.031 |
| YBX3 | NK cells | 0.090 | 0.038 |
| YBX3 | iDC | -0.079 | 0.070 |
| YBX3 | T helper cells | -0.071 | 0.103 |
| YBX3 | Tem | 0.068 | 0.120 |
| YBX3 | Tcm | 0.054 | 0.216 |
| YBX3 | NK CD56 bright cells | -0.050 | 0.254 |
| YBX3 | Mast cells | -0.045 | 0.303 |
| YBX3 | B cells | -0.037 | 0.398 |
| YBX3 | CD8 T cells | -0.024 | 0.583 |
| YBX3 | NK CD56dim cells | -0.022 | 0.619 |
| YBX3 | DC | 0.020 | 0.651 |
| YBX3 | Cytotoxic cells | 0.017 | 0.696 |
| YBX3 | T cells | -0.017 | 0.699 |
| YBX3 | Neutrophils | -0.009 | 0.840 |
