# Supplementary material for: Identification of RNA-binding protein YBX3 as an oncogene in clear cell renal cell carcinoma
Source: Funct Integr Genomics. 2023 Jul 7;23(3):225. doi: 10.1007/s10142-023-01145-6 (PMC10329074; doi:10.1007/s10142-023-01145-6)
Supplement: Supplementary file 2 — Supplementary file2 (DOCX 19 KB) [file 10142_2023_1145_MOESM2_ESM.docx]

Table S2 Statistical difference for survival analysis of YBX3 expression in TCGA-KIRC samples.

Method chi square HR CI p value

Log-rank 31.23 2.29 1.64-3.19 0.000

Cox regression 2.29 1.70-3.09 0.000
